# Biogenesis, cellular effects, and biomarker value of circHIPK3

**DOI:** 10.1186/s12935-021-01956-2

**Published:** 2021-05-11

**Authors:** Yihan Fu, Hong Sun

**Affiliations:** 1 Obstetrics and Gynecology Hospital, Fudan University, Shanghai, China; 2 Shanghai Key Laboratory of Female Reproductive Endocrine Related Diseases, Shanghai, China

**Keywords:** CircHIPK3, MiRNAs sponge, Biomarker, Cancer

## Abstract

Competing endogenous RNAs (ceRNAs) can indirectly regulate gene expression by competitively binding to microRNA(miRNA) through miRNA response elements (MREs) to affect miRNA-induced gene regulation, which is of great biological significance. Among them, circular RNA (circRNA) has become a hotspot due to its highest binding capacity. A specific circRNA discussed in this review, circHIPK3, has been studied for its biological characteristics, function, cellular effects and its relationship with tumors and various diseases. Here, we review the recent researches about circHIPK3 in detail and aim to elucidate accurate conclusions from them. These circHIPK3-miRNAs-mRNA pathways will further advance the application of circHIPK3 in diseases development, early diagnosis and gene targeting therapy.

## Background

Multiple studies have uncovered the biogenesis, features, functions, and relationships with diseases of circHIPK3 as a ceRNA. Here, we reviewed the recent researches about circHIPK3 in detail and aimed to elucidate accurate conclusions from them.

## Main text

CircRNA (circRNA) is a class of endogenous non-coding RNA molecules lacking 5′ and 3′ terminals [5]. Now, with the emergence and easy access to large-scale sequencing databases, recent works have revealed that circRNAs were characterized by conserved, endogenously abundant, and stable in mammalian cells [30]. Due to special structure, they cannot be degraded by acid endonucleases [40], thus having a longer half-life and more stable pre-transcriptional regulatory function [55]. There is growing evidence that they play vital regulatory roles in a number of biological processes and disease occurrences.

**Fig. 1:**
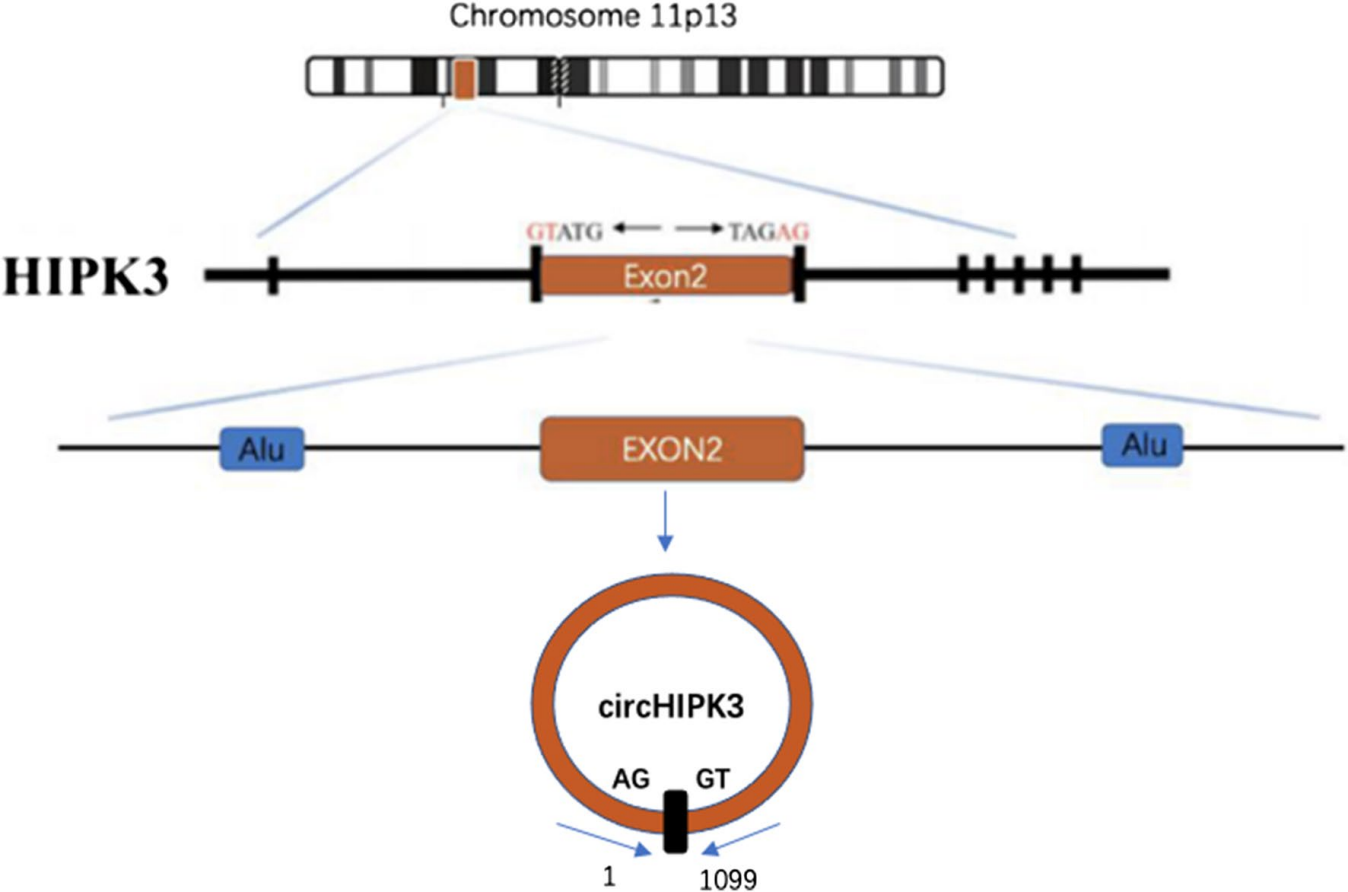
Biogenesis and formation of circHIPK3

HIPK3, the homeodomain-interacting protein kinase 3 gene, is located on chromosome 11p13 [83]. Genome sequence indicates that the second exon from HIPK3, coupled with the long flanking introns with complementary Alu repeats on both ends, promotes its cyclic characteristic (Fig. 1) [43, 101]. It’s product, hsa_circ_0000284, also named circHIPK3, which has been studied widely [89], is preferentially localized in the cytoplasm, stably and abundantly expressed in different human tissues, such as the lung, heart, stomach, colon, and brain [101]. In studies to date, it was observed that circHIPK3 can be transferred through exosomes between donor cells and recipient cells as a messenger to mediate multiple signaling pathways for cellular communication [25]. Either in the cytoplasm or exosome, dysregulation of circHIPK3 engaged in various pathological processes, drug responses and so on.

**Fig. 2:**
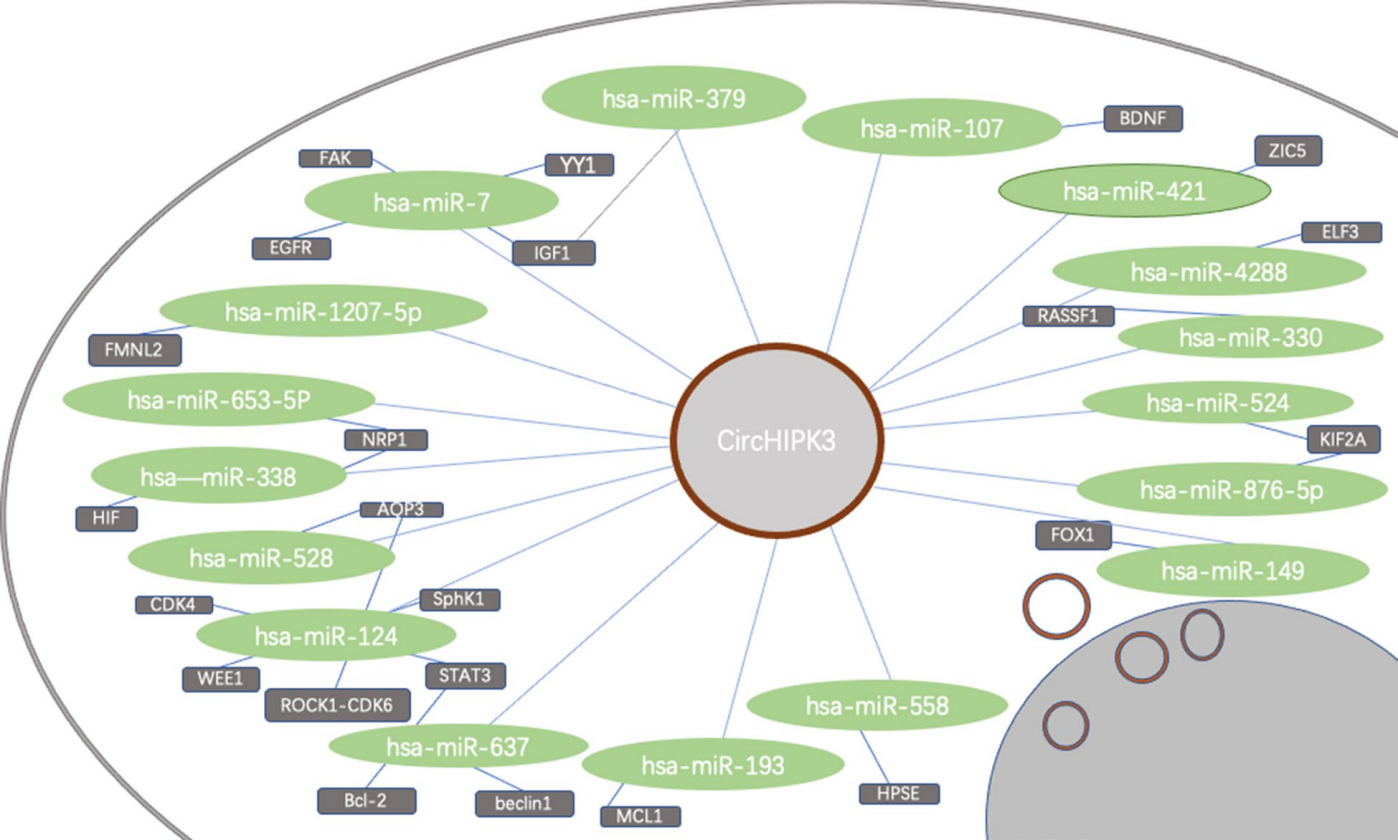
Overview of circHIPK-sponged miRNAs and their targeted genes

Circular RNA is known to perform its cellular effects by playing the following four roles: microRNA sponge, regulators of transcription and splicing, adaptor for protein–protein interaction and ribosomal RNA processing. Among which, multiple studies have revealed the circular RNA, as ceRNA, indirectly regulated gene expression by competitively binding different miRNAs and blocking miRNAs from binding to their target mRNAs, thereby downregulating the inhibitory functions of miRNAs on their target mRNAs (Fig. 2) [101]. For instance, by sponging miR-124, the most studied miRNA it interacts with, circHIPK3 induces tumor proliferative and invasion in a variety of tumors including breast, prostate, glioma, oral squamous cell carcinoma, gallbladder, osteosarcoma and lung carcinoma [28, 33, 84, 85, 94] Contrary to most cases, when circHIPK3 sponges miR-558, it causes an opposite effect that inhibits angiogenesis, migration and invasion [41]. Similarily, by adsorbing different miRNAs, circHIPK3 can have a completely different effects on drug sensitivity, too. For instance, the elevated level of circHIPK3 can promote gemcitabine (GEM) resistance in pancreatic cancer cells by targeting RASSF1 via miR-330-5p [52], while the overexpression of circHIPK3 in bladder cancer (BC) leads to GEM sensitivity [86].

## Cellular effects caused by circHIPK3

### Proliferation

Circular RNA profiling reveals that circHIPK3 expression is dynamically regulated in various cancers, and its expression level is correlated with tumor progression to some extent [83]. Pearson’s correlation analysis done by Zhu et al. unveiled the expression of circHIPK3 positively correlates with Ki-67 (a proliferation marker), which further supported that circHIPK3 played a fundamental role in cancer progression by regulating proliferation [103]. Other than cancer cells, it also influences the physiological behavior of normal cells. For example, it was detected to affect the proliferation of mammary epithelial cells in dairy cows though STAT5 signaling pathway [46]. CircHIPK3 regulates cell proliferation in a variety of ways and directions, such as by accelerating G2/M transition [45], or by inhibiting apoptosis [84]. Although it promotes proliferation in most conditions, it can exert an opposite effect in bladder cancer [41].

### Autophagy

Autophagy is an evolutionarily conserved intracellular process that maintains homeostasis by degrading dysfunctional organelles and providing energy for cells [54]. It was found that autophagy was significantly upregulated in cancer, since tumor cells depend on it for survival under ischemic or various stresses [1]. Chen et al. suggested that the ratio between circHIPK3 and linear HIPK3 (C/L value) reflected the levels of autophagy to some extent and a high C/L value (> 0.49) was an index of poor prognosis, particularly in advanced-stage patients [13]. It was observed in STK11 mutant lung cancer cell lines that autophagy caused by deletion of circHIPK3 would lead to inhibition of cell proliferation [13]. CircHIPK3-mediated autophagy can also induce oxaliplatin-resistance in colorectal cancer [100]. Interestingly, autophagy induced by forced expression of circHIPK3 can reduce lipid content in high fat diet (HDF) mice and ox-LDL treated human umbilical vein endothelial cells(HUVECs) through miR190/ATG7, indicating that it plays an essential role in the pathogenesis of atherosclerosis and provides a targeted idea for it [82].

### Angiogenesis

Angiogenesis is a key process in tumor metastasis, mainly through the formation of new blood vessels around solid tumors to provide oxygen, nutrients and dispose wastes [89]. circHIPK3 can accelerate angiogenesis and cancer metastasis by modulating vascular endothelial growth factor (VEGF), heparanase (HPSE) and other molecules [65]. Besides, this pathological process can also aggravate the progression of other diseases by mediating vascular dysfunction. For example, by blocking the function of miR-30a, it can worsen diabetic retinopathy via the regulation of angiogenesis, proliferation and inflammatory response of retinal endothelial cells [66]. Meantime, studies have shown that cardiomyocytes(CMs) subjected to hypoxia could promote angiogenesis by releasing circHIPK3-rich exosomes, thereby reducing infarction size and alleviating oxidative stress injury, which provides a novel solution for post-MI management [80].

### EMT/FMT

Epithelial-mesenchymal transition (EMT) plays a crucial role in tumor metastasis, which refers to the process in which cells acquire invasion and metastasis ability due to loss of epithelial characteristics [70]. Weiwei Qian et al. found that circHIPK3 can act as a ceRNA of miR-338-3p to regulate HIF-1α mediated EMT in cervical cancer (CC) [63]. Furthermore, circHIPK3 plays a regulatory role in metastasis in hepatocellular carcinoma (HCC) and glioma by sponging miR-338-3p and miR-124-3p, respectively. Then it induces the upregualation of EMT-associated proteins E-cadherin, N-cadherin, vimentin, and ZEB2, thus promoting EMT [39, 87–89].

Meanwhile, myofibroblasts predominantly emerging through fibroblast-to-myofibroblast transition (FMT) are considered to be the crucial collagen-producing cells in pulmonary/cardiac fibrosis [59]. Zhang et al. came up with that circHIPK3 was significantly upregulated in bleomycin-induced pulmonary fibrosis mouse models, silencing which could inhibit FMT and reverse the proliferation of fibroblasts [99].

### Differentiation

Cell differentiation refers to the process of cells from the same origin gradually transforming into cell populations with different morphological structures and functional characteristics. The association between circRNAs and cell differentiation has also received extensive research attention [19]. At present, we only have a preliminary understanding about circHIPK3 and differentiation. Yao et al. found that circHIPK3 played a vital role in skeletal muscle’ differentiation by absorbing miR124 or miR379 [9, 92]. CircHIPK3 could induce the upregulation of Th2 cell-specific transcription factor GATA-3 by regulating its target miR-495, thereby promoting ovalbumin-induced Th2 differentiation, which lead to aggravated nasal symptoms in asthmatic model (AS) mice. They also found that nasal symptoms in AR mice could be relieved by intranasal administration of circHIPK3 [104].

### Cytotoxic

CircHIPK3 can also act as a mediator for cell injury in different microenvironments. For example, downregulation of circHIPK3 mediates oxidative damage induced by H_2_O_2_ in human osteoblasts, including decreased cell viability and cell death, which can be alleviated by lentiviral overexpression it [44]. Study carried out by Jin et al. demonstrated that circHIPK3 could be viewed as an oncogene of gastric cancer under long-term hypoxia microenvironment, and it could promote gastric cancer metastasis through miR-653-5p/miR-338-3p-NRP1 axis under long-term hypoxia stimulation [32]. Dexamethasone (DEX)-induced cytotoxicity in human osteoblasts is also mediated by circHIPK3 downregulation. Congya Zhu et al. found out that ectopic overexpression of circHIPK3 could effectively inhibit DEX-induced apoptosis and programmed necrosis [102]. Ying et al. came up with that circHIPK3 mediated vascular endothelial cell injury in the high glucose microenvironment. Therefore, targeting circHIPK3-miR-124 pathway might be a novel approach to alleviate diabetes-related vascular injury [8].

### Pyroptosis

Unlike apoptosis, a programmed process that results in non-inflammatory cell death, pyroptosis, or caspase 1-dependent cell death, is inherently inflammatory triggered by multiple pathological stimuli, such as heart attack or cancer, and is essential for controlling microbial infections [3]. Prior results revealed that pro-inflammatory cytokines such as IL-1β were released during pyroptosis in acute myocardial infarction [56]. Followed by, further exploration reported that human umbilical cord mesenchymal stem cells-derived exosomes (UMSC-Exo) could prevent ischemic injury by releasing circHIPK3, which in turn down -regulated miR-421, leading to the inhibition of pyroptosis and release of IL-1β and IL-18 [90]. Pyroptosis, the main mode of acinar cell death during acute pancreatitis (AP), can aggravate the inflammatory response by releasing cellular contents and inflammatory factors such as tumor necrosis factor alpha (TNF-α) and IL-1β [27]. It was also observed that circHIPK3 promoted pyroptosis in acinar cells via miR-193a-5p/GSDMD axis during AP, which eventually worsened the condition [76].

Above, we briefly introduced the cellular effects related to circHIPK3 and briefly summarized molecular mechanisms by which it played roles in the occurrence and development of various pathological processes. These findings provide new ideas for targeted treatment of related diseases as an understudied and important potential next frontier.

## CircHIPK3 as biomarkers

Since its stable expressed, cell/tissue-dependent and stage-specific character, several of circRNAs are currently considered to be relatively mature biomarkers for disease diagnosis [5]. So here, we’d like to introduce circHIPK3's association with various diseases by adsorbing different miRNAs, and evaluate its value as diagnostic markers.

### Malignant tumor

CircHIPK3 has been studied most extensively and deeply in tumors. It has been involved in tumor progression mainly through the following three aspects: cancer growth, metastasis, and angiogenesis [89]. Researches have shown that its level is correlated with tumor volume, tumor and Lymph Node and Metastasis (TNM), as well as patients’ survival in various cancers [81, 89].

#### As a tumor promotor

By competing for tumor suppressive miRNAs and their target oncogenes, circHIPK3 facilitates proliferation, migration and invasion in various cancers (Table 1). In the following, we give a brief introduction of its role as a tumor promotor.

**Table 1:** Different functions when circHIPK3 sponges different miRNAs in cancers

Roles	Cancers	miRNA	mRNA/protein	Function	Refs.
Tumor promotor	Non-small cell lng cancer	miR124-3p	SphK1, CDK4	Proliferation	[94]
			STAT3-PRKAA/AMPKalpha	Proliferation, migration, invasion, autophagy	[13]
		miR-149	FOXM1	growth, apoptosis, migration, invasion	[53]
		miR-379	IGF1	Proliferation	[73]
	Colorectal cancer	miR-637	STAT3/Bcl-2/beclin1	Autophagy, oxaliplatin-resistance	[100]
		miR-7	FAK, IGF1R, EGFR, YY1	Proliferation, migration, invasion, apoptosis	[95]
		miR-1207-5p	FMNL2	Proliferation, migration, invasion	[91]
	Cervical cancer	miR-338-3p	HIF-1	Growth, metastasis, EMT	[63]
	Gallbladder cancer	miR-124	ROCK1-CDK6	Proliferation, apoptosis	[33]
	Glioma	miR-124-3p	STAT3, WEEE1	Proliferation, invasion, EMT	[47, 85]
			CCND2	Proliferation, invasion	
		miR-654	IGF2BP3	Proliferation, invasion, tumor propagation	[31]
		miR-421	ZIC5	Invasion, apoptosis temozolomide (TMZ) resistance	[24, 25]
		miR-524-5p	KIF2A	Apoptosis, proliferation, metastasis, Temozolomide resistance	[93]
	Prostate cancer	miR338-3p	Cdc25B/Cdc2	G2/M transition, proliferation, apoptosis	[45]
			ADAM17	Proliferative, invasive	[6]
		miR-193a-3p	MCL1	Proliferation, migration, invasion	[10]
	Oral squamous cell carcinoma	miR-124		Proliferation	[77]
		miR-381-3p	YAP1	Proliferation, invasion, migration, apoptosis	[4]
	Nasopharyngeal carcinoma	miR-4288	ELF3	Proliferation, migration, invasion	[34]
	Hepatocellular carcinoma	miR124	AQP3	Proliferation, migration	[12]
		miR-582-3p	AQP3	Proliferation, migration, invasion, apoptosis	[97]
	Pancreatic cancer	miR-330-5p	RASSF1	Proliferation, invasive, migration, apoptosis, promotes Gemcitabine Resistance	[52]
	Breast cancer	miR-193a	HMGB1-PI3K-AKT	Proliferation, migration, invasion	[15]
				Trastuzumab resistance	[98]
	Chronic myeloid leukemia				[21]
Tumor suppressor	Bladder cancer	miR-558	HPSE	Migration, invasion, angiogenesis	[41]
				Attenuate gemcitabine resistance	[87]

### Non-small cell lung cancer

Lung cancer is the leading cause of cancer-related deaths worldwide, more than 85% of which are now non-small cell lung cancer (NSCLC), with a projected 5-year survival rate of 15.9% [14]. Chen et al. demonstrated that the ratio of circHIPK3 to linear HIPK3 (C:L ratio) > 0.49 could be used as a biomarker of poor prognosis, especially for advanced NSCLC patients, [13].

CirHIPK3 can act as an oncogene in NSCLCs by sponging multiple miRNAs. Lu H et al. found that circHIPK3 regulates FOXM1 expression by sponging miR-149 [53]. Yu et al. demonstrated circHIPK3 could silence miR-124, resulting in increased expression of its downstream proteins, including Sphk1, CDK4, and STAT3 [94]. Feng Tian et al. verified that circHIPK3 could act as an oncogenic gene of NSCLC through miR-379/IGF2BP3 signaling pathway [73]. These observations offer a footstone for molecular-targeted treatment for NSCLCs patients.

### Glioma

Glioma is a malignant tumor that often occurs in adult brain. Approximately 80% of glioma tumors are aggressive and highly recurrent [74]. It was found that circHIPK3 was abundant in human glioma cancer tissues. Overexpression of circHIPK3 was linked to poor prognosis [31] and resistance to Temozolomide(TMZ) [24, 25].

CircHIPK3 could affect tumor progression through miR-654/IGF2BP3 pathway [31]. Elevated expression of circHIPK3 could reverse miR-124-induced malignant behavior via elevating the level of its targeting downstream molecules such as ROCK1, CDK6, WEE1 and STAT3 [28, 33, 89].These studies not only highlight the promoting role of circHIPK3 in the pathogenesis of glioma, but also make circHIPK3 a novel biomarker molecule due to its stability and enrichment.

### Colorectal cancer

Colorectal cancer(CRC) is the third most common cancer in the Western hemisphere and the incidence increases with age [26]. Furthermore, circHIPK3 may also have strong possibility to be a prognostic biomarker in CRC. It was found that expression of circHIPK3 was positively correlated to clinical stage and distant metastasis. Multivariate Cox’s analysis indicated that increased circHIPK3 was an independent prognostic factor in CRC [95]. Consistently, a spearman correlation analysis performed by Yan et al. detected that elevated circHIPK3 was significantly related to poor clinicopathological features as tumor(T) classification (p = 0.010), nodal (N) classification (p = 0.004), metastasis (M) classification (p = 0.010) and liver metastasis (p = 0.024) in CRC [91].

As for its regulation network, studies revealed that circHIPK3 boosted CRC cell proliferation and invasion by sponging miR-1207-5p and regulating FMNL2 expression [91]. Knockdown of circHIPK3 impeded growth and metastasis of CRC in xenograft tumor model, which further supported that targeting the c-Myb/circHIPK3/miR-7 axis might be a promising treatment approach for CRC patients [95].

### Prostate cancer

Prostate cancer PCa is one of the most common cancers in men. It becomes incurable once the tumor has metastasized [57]. CircHIPK3 play a significant role in tumor occurrence and progression in PCa. It was estimated that high circHIPK3 expression was correlated with advanced tumor stage [10, 11], and high Gleason scores [45].

CircHIPK3 acts as a competing endogenous RNA of miR-338-3p to promote cell growth and metastasis in PCa, via regulating Cdc25B/Cdc2 mediated G2/M transition [6, 48]. CircHIPK3 also promoted MCL1-mediated proliferation and metastasis via decoying of miR-193a-3p. Moreover, circHIPK3 knockdown could suppress PCa growth in vivo [10]. Taken together, circHIPK3 may be a potential biomarker of therapeutic and diagnosis in PCa.

### Hepatocellular carcinomas

Hepatocellular carcinoma (HCC) is the most common type in the sub-classification of liver cancer, and presents a serious threat to global health. CircHIPK3 may play an oncogenic role in HCC. It was suggested that the level of circHIPK3 was associated with tumor differentiation (*p* = 0.028) and TNM stage (*p* = 0.029) [12].

With sufficient validation in vivo, Chen et al. confirmed the notion that circHIPK3 promoted AQP3-mediated proliferation and metastasis via decoying of miR-124 [12]. Hongbin Zhang et al. demonstrated circHIPK3 facilitated HCC progression by mediating miR-582-3p/DLX2 pathway [97]. These results provide a novel insight into the role of circHIPK3 in the progression and treatment of HCC.

### Cervical cancer

circHIPK3 facilitated cervical cancer (CC) progression through protecting HIF-la from miR-338-3p‐mediated silencing, which supported that the circHIPK3/miR-338-3p/HIF-1 alpha axis might be a novel target for CC [63].

### Nasopharyngeal carcinomas

Nasopharyngeal carcinomas (NPC) patients with a higher level of circHIPK3 indicated a lower overall survival (OS) and metastasis-free survival (MFS), meaning that it might be a prognostic indicator in NPC patients [34].

### Gallbladder cancer

Ding Kai et al. detected that circHIPK3 expression is increased in gallbladder cells compared with healthy control samples, and its overexpression is associated with proliferation and apoptosis. Blocking circHIPK3 can decrease ROCK1-CDK6 expression and leads to reduced progression of this cancer. They suggested that circHIPK3 could be a promising prognostic biomarker in gallbladder patients and may be considered as an appropriate therapeutic target for reducing ROCK1-CDK6 activity in these patients [33].

### Pancreatic cancer

Due to rapid progression, the highly aggressive tumor phenotype, and resistance to chemotherapy, pancreatic cancer (PC) has an extremely poor prognosis. Despite ongoing research efforts and significant advances in the treatment of the disease over the past few decades, the clinical outcome of PC has improved only with minor overall changes in survival after initial diagnosis. The oncogenic effect of circHIPK3 on proliferation and invasion of PC cells has been reported by Liu et al., moreover, they came up with that circHIPK3 could also foster gemcitabine (GEM) resistance in PC cells by targeting RASSF1 via miR-330-5p [52]. Hence, studies have laid the groundwork for future therapeutic strategies for PC, especially for GEM-resistant PC.

### Renal carcinoma

It has been suggested that circHIPK3 could be a valuable prognostic biomarker in renal carcinoma (RC) patients and may be considered as an appropriate therapeutic target, since the high expression of circHIPK3 was closely correlated with a high TNM grade (P = 0.001), lymph node metastasis (P = 0.037), distant metastasis (P = 0.022), a bigger tumor size (P = 0.021) and a higher Fuhrman grade (P = 0.004). Upregulation of circHIPK3 can result in a more aggressive phenotype in A498 and 786-O Cells by decoying miR-508-3p expression and further activation of CXCL13 oncogenes [24, 25]. Consistently, research done by Lai, J revealed that patients with low expression of circHIPK3 in the cytoplasm of RC tissues and cells had higher survival rates than those with high expression. The oncogenic effect of circHIPK3 on RC cells by targeting miR‐485-3p signaling pathway has also been reported by them [35].

### Oral squamous cell carcinoma

Oral squamous cell carcinoma (OSCC) represents an increasing problem in the global public health, and is the most frequent malignancy in oral cavity and 1 of the 10 most common cancers worldwide [61]. Therefore, finding biomarkers to improve the diagnosis, prognosis, and treatment of this cancer is essential. Wang et al. found that the expression of circHIPK3 was closely associated with its TNM stage (*P* < 0.05) and tumor grades (*P* < 0.05), and its increased expression led to promoted proliferation, invasion and migration through miR‐124 blockage [77]. Also, in a recent study, it was shown that circHIPK3 could stimulate growth and development in OSCC cells through sponging and directly inhibiting of miR-381-3p. It thus could increase Yes-associated protein1 (YAP1) expresion, indicating a promising therapeutic strategy for OSCC [4].

### Breast cancer

Breast cancer (BC) is the most frequently diagnosed cancer in women and ranks second among causes for cancer related death in women. Evidence in literature has shown that the past and ongoing research has an enormous implication in improving the clinical outcome in BC. However, poor prognosis and drug resistance especially in triple-negative breast cancer (TNBC) present major dilemmas we face [20]. Therefore, there is a need for exploring new biomarkers to classify patients and provide them with the most appropriate treatments. A study on BC pointed to the important clinical and functional role of circHIPK3 as an oncogenic noncoding RNA and a potential biomarker for therapeutic targets in BC. Increased circCHIPK3 expression was found to be positively associated with advanced TNM stages (III–IV stages) (*P* = 0.014) and lymph node metastasis (*P* = 0.001). Kaplan‐Meier analyses revealed that the high circHIPK3 expression group had poorer survival than the low group (*P* = 0.034).They also pointed out that its increased expression inhibited the regulatory effect of miR‐193a on malignant-promoting pathways such as HMGB1/PI3K/AKT [15]. According to recent research, knockdown of circHIPK3 may help overcome trastuzumab chemotherapy resistance due to long-term use [105].

### Chronic myeloid leukemia

Chronic myeloid leukemia (CML) is a stem cell disorder characterized by unrestricted proliferation of the myeloid series that occurs due to the BCR-ABL fusion oncogene as a result of reciprocal translocation t(9;22) q34;q11). Imatinib gave encouraging results both in case of safety as well as tolerability profile. However, about 2–4% of patients show resistance and mutations have been found to be one of the reasons for it [58]. Previous results have shown that circHIPK3 was significantly upregulated in peripheral blood mononuclear cells (PBMC) and serum samples from CML compared with healthy controls. Multivariate analysis revealed that the serum circHIPK3 level (p = 0.02) was an independent factor for predicating the prognosis of CML patients. And its level was associated with Sokal relative risk (p = 0.017), but not BCR/ABL mutant status. CircHIPK3 overexpression reduced the inhibitory effect of miR‐124 on its B4GALT1 target gene, activated the p65 signal in an NF‐kB dependent manner, thus fostering cell proliferation and inhibiting apoptosis of CML cells [21]. Underlying the difficulties of drug resistance in current CML treatment, we suggested that more functional experiments should be conducted to better understand its molecular mechanism and more samples should be collected to explore whether it has the potential to be a biomarker of drug resistance.

#### As a tumor suppressor

Although most evidence suggests that circHIPK3 has a cancer-promoting effect, some studies have revealed its opposite effects, and there have even been instances of contradictory effects coexisting within the same tumor (Table 2). Taken together, it could be generalized that the effect that it exerted was largely determined by the downstream miRNA it attached.

**Table 2:** circHIPK3 expression level is altered in some tumors

Cancer	Contradictory results	miRNA	Refs.
Osteosarcoma	Upregulated	miR-637/STAT3	[29]
	Downregulated		[86]
Epithelial ovarian cancer	Upregulated		[48]
	Downregulated		[72]
Gastric cancer	Upregulated	miR107/BDNF	[81]
		miR-876-5p	[38]
		miR-653-5p	[32]
		miR-338-3p	
	Downregulated		[22]

### Bladder cancer

circHIPK3 is significantly down-regulated in bladder cancer (BC) tissues and cell lines, and negatively correlates with its grade, invasion as well as lymph node metastasis. Mechanistic studies revealed that circHIPK3, containing two critical binding sites for the miR-558, suppressed BC proliferation by mediating miR-558/HPSE pathway.

### Gastric cancer

Gastric cancer (GC) is a common malignant tumor of digestive tract. Due to the lack of effective diagnostic markers and therapeutic targets, the prognosis is often poor due to tumor metastasis and recurrence [75]. By performing circRNA microarray, Wei et al. found circHIPK3 was upregulated in all GC tissues and cells tested, and might exert a significant regulatory role in GC [81]. What’s more, higher expression was observed in infiltrative-type GC cells than in expanding-type GC cells [16]. Furthermore, Wei et al. detected that circHIPK3 levels were associated with Tumor and Lymph Node and Metastasis (TNM) stage (P = 0.032) [81]. Analyzed by the Kaplan–Meier method, Liu et al. revealed that the expression level of circHIPK3 was negatively correlated to the overall survival of GC patients [50]. The bioinformatics analyses done by Cheng J et al. demonstrated that expression of circHIPK3 was associated with Ming’s classification in GC [16]. To sum up, researches has verified circHIPK3’s value as biomarkers for classification and prognosis in GC.

CircHIPK3 exerts oncogenic properties in GC via suppression of multiple miRNAs, for instance,miR-124,miR-29b,miR-876-5p, miR-653-5p,miR-338-3p,miR-107 [16, 32, 81], as well as the Wnt/β-catenin pathway [50], targeting expression of which can be a potential treatment strategy for GC. However, Ghasemi and colleagues demonstrated that the level of circHIPK3 was significantly downregulated in GC and its expression was correlated with age and M classification [22]. Therefore, more samples need to be tested and compared to draw more objective conclusions.

### Ovarian cancer

Present studies about circHIPK3 in ovarian cancer (OV) varied widely. In Teng et al.’s study, qRT-PCR and expression profiles both confirmed that circHIPK3 was stably under-expressed in OV epithelial cell lines, silencing it promoted OV progression [72]. However, Liu et al. found that circHIPK3 was an onco-promoter of OV and its expression was associated with lymph node infiltration, FIGO staging, DFS, and OS [48].

### Osteosarcoma

Same situation occurs in Osteosarcoma (OS). Huang et al. suggested the expression of circHIPK3 in OS cell lines was significantly upregulated [29], while Xiao-Long et al. raised that circHIPK3 is stably down-regulated in the OS cell lines, tissues and plasmas than the corresponding controls [86].

To put in a nutshell, the contradictory expression of circHIPK3 within the same type of tumor above suggests that further and repeated verification is needed. In fact, this phenomenon also presented in another widely studied circRNA, CDR1as. As previously described, the alters in CDR1as expression were not consistent to a single disease, either. Some authors thus speculated that the regulatory effect of CDR1as was extensive but not specific [23]. We hereby support this view, and would like to come up that the degree of abnormal circHIPK3 expression, whether down-regulated or up-regulated, is estimable for determining the degree of malignancy in a large number of tumors.

### Other diseases

#### Diabetes

In addition to cancer, circHIPK3 is also associated with many diseases. Its role in diabetes has been investigated mostly [71]. circHIPK3 was found to be reduced in the islets of diabetic mice. Mimicking this decrease in the islets of wild type animals resulted in impaired insulin secretion, reduced β-cell proliferation, and survival. Transcriptomic analysis revealed that, by sequestering miR-124-3p and miR-338-3p, circHIPK3 could regulate the expression of key β-cell genes, such as Slc2a2, Akt1 and Mtpn [71]. According to Cai et al., circHIPK3 participated in diabetes-related metabolic disorders characterized by hyperglycemia and insulin resistance by sponging miR-192-5p and up-regulating FOXO1 [7].

Moreover, dysregulation of circHIPK3 is also closely associated with the progression of various diabetes complications, which is mainly related to endothelial cell injury induced by high glucose (HG) [8, 67]. For instance, diabetic nephropathy (DN) is a major cause of end-stage renal disease throughout the world [62]. By establishing the HG-induced HK-2 cells as models to mimic renal epithelial cells damage of DN, Langen Zhuang et al. suggested that overexpression of circHIPK3 can alleviate HG toxicity to human renal tubular epithelial cells through miR-326/miR-487a-3p/SIRT1 pathways [105]. However, Liu et al. came up with that circHIPK3 increased significantly under HG in rat mesangial cells(MCs). And it mediated the occurrence and development of diabetic nephropathy by acting as ceRNA of miR-185 [49], silencing of which could reverse the effects on proliferation and mRNA abundance of cyclin D1, PCNA, TGF-β1, Col. I, and FN in MCs. Furthermore, Shan et al. found that circHIPK3 expression was significantly higher in the fibrovascular membrane of diabetic patients than in the idiopathic anterior retinal membrane of control patients. In addition, circHIPK3 was significantly upregulated in plasma and aqueous humor of diabetic patients [68]. In vivo silencing of circHIPK3 can reverse capillary proliferation, inhibit inflammation, and reduce retinal vascular dysfunction [66]. However, they did not elaborate the relationship between circHIPK3 content with the grade of diabetic retinopathy, which is worth further exploration.

Additionally, it has also been found that circHIPK3 is involved in diabetic cardiomyopathy (DCM) by upregulating the expression of COL1A1 and COL3A1 via inhibiting miR-29b-3p. CircHIPK3 knockdown could effectively reduce myocardial fibrosis and ameliorate cardiac function in DCM mice [79].

What’s more, neuropathic pain is one of the most common complications of diabetes, which seriously affects the quality of life of patients. CircHIPK3 was upregulated in the serum of diabetic patients with neuropathic pain, and it was positively correlated with extent of neuropathic pain. Wang et al. presented the first evidence that intrathecal circHIPK3 shRNA treatment could be used to treat neuropathic pain in vivo [78].

#### Myocardial infarction

Roles of circHIPK3 on myocardial cells after myocardial infarction (MI) are more complex. Xiaoyun Si et al. showed that circHIPK3 overexpression alleviated cardiac dysfunction and deduced fibrosis area after MI by inducing myocardial regeneration and coronary angiogenesis [69]. Moreover, circHIPK3-rich exosomes released by cardiomyocytes under hypoxia induced VEGFA expression by inhibiting miR-29a activity, thereby accelerating cell cycle progression, reducing infarction area, promoting angiogenesis around the infarction area, and regulating oxidative stress injury after MI (Liu et al. 2020). However, the findings of Bai et al. were contradictory to the above results. Their experimental results showed that the highly expressed circHIPK3 in the myocardial ischemia–reperfusion injury model could aggravate myocardial ischemia–reperfusion injury by binding with miR-124-3p, up-regulating pro-apoptotic Bax and down-regulating anti-apoptotic Bcl-2, [2]. Deng et al. [18] has provided a more comprehensive explanation about the role of circHIPK3 post-MI. They showed a positive feedback between circHIPK3 and adrenaline. CircHIPK3 significantly increased after MI can reverse heart failure in the short term by enhancing adrenaline through the activation of β-adrenergic receptor (β-AR). However, in the long term, overactivation of β-AR can lead to the disintegration of the RyR2 subunit calstabin2 or decrease the activity of SERCA2a, thus interfering with the Ca^2+^ circulation and ultimately causing heart damage [37].

#### Cardiac/pulmonary fibrosis

It was found that circHIPK3 expression was significantly increased in patients with heart failure after Ang II treatment and was associated with cardiac fibrosis, which could be effectively reduced by silencing Ang II. Thus, they provided a promising approach for the treatment of Ang II-induced cardiac fibrosis [60].

There was also a specific high expression of circHIPK3 in patients with idiopathic pulmonary fibrosis. Further studies showed that circHIPK3, as a miR-338-3p sponge, could induce fibroblast to myofibroblast (FMT), revealing a potential target gene for the treatment of pulmonary fibrosis [99].

#### Osteoarthritis

Q, Wu et al. detected that circHIPK3 was significantly up-regulated in osteoarthritis (OA) cartilage tissues and cells by RT-PCR. Silencing circHIPK3 can inhibit the expression of SOX8 through miR-124, thereby promoting the apoptosis of OA chondrocytes. The molecular mechanism of circHIPK3 in this study is expected to provide new ideas for the treatment of OA [84].

#### Age-related cataract

CircHIPK3 was dramatically down-regulated in age-related cataract (ARC), silencing which, but not HIPK3 mRNA, could significantly accelerate apoptosis upon oxidative stress and inhibit EMT by targeting the miR-193a/CRYAA axis, as well as miR-221-3p/PI3K/AKT pathway, providing a novel insight into the pathogenesis of ARC [17, 51].

#### Acute pancreatitis

Acute pancreatitis (AP), especially severe acute pancreatitis (SAP), is an inflammatory disorder with high morbidity and mortality [36]. According to Wang et al.’s research, circHIPK3 promotes pyroptosis, the main mode of acinar cell death during AP through regulation of the miR-193a-5p/GSDMD axis, which eventually aggravates AP. Additionally, the level of circHIPK3 in SAP patients was significantly higher than that in mild AP(MAP) patients, suggesting that the expression of circHIPK3 is associated with severity of the disease [77]. As we known, SAP runs a biphasic course. In the first 1–2 weeks, proinflammatory responses lead to systemic inflammatory response syndrome (SIRS). If SIRS goes severe, it can lead to early multi-system organ failure (MOF). Subsequently, during the transition from pro-inflammatory to anti-inflammatory responses, patients are at risk of intestinal microbiota translocation and secondary infection of necrotic tissue, which may lead to sepsis and advanced MOF [96]. Consequently, it is essential to make an informed identification of the course of SAP we may explore ulteriorly whether circHIPK3 can be used as a biomarker to distinguish these two stages and provide guidance for SAP nursing and management of complications.

### Chemoresistance

Targeting circHIPK3 is a novel choice to solve drug resistance. CircHIPK3 was obviously increased in Temozolomide (TMZ)-resistant glioma cells and their exosomes, knockdowning of which facilitates Temozolomide sensitivity by regulating miR-421/ZIC5 axis and miR-524-5p/KIF2A-mediated PI3K/AKT pathway [24, 25, 93]. Therefore, circHIPK3 may be the potential target for the diagnosis and therapy of TMZ-resistant cancer. What’s more, circHIPK3 leads to drug resistance via miR-637/STAT3/Bcl-2/ Beclin1 pathway, and can be used to predict the prognosis of CRC patients with oxaliplatin-based chemotherapy [100]. Interestingly, the pharmacodynamic effects of circHIPK3 on gemcitabine (GEM) vary according to it's downstream miRNAs. CircHIPK3 fostered GEM resistance in pancreatic cancer cells via miR-330-5p/RASSF1 axis [15]. Surprisingly, circHIPK3 also acts as a drug sensitizer. The low-expression of circHIPK3 contributed to insensitivity to gemcitabine in bladder cancer patients [89]. It's detected that circHIPK3 might be a potential biomarker and an ideal therapeutic target for heart failure (HF) [69]. What’s more, it can also be a helper for adrenaline in the HF treatment through miR-17-3p—ADCY6 axis, but was harmful for heart in the long run [18].

## Conclusion

In this review, we provide an overview of one circular RNA, circHIPK3, acting as a sponge for miRNAs to block them from binding to their target mRNAs, regulates gene expression in the post-transcriptional. Hereafter, the role of circHIPK3 in cell proliferation, autophagy, differentiation, pyroptosis, EMT regulation, cytotoxic and angiogenesis is discussed. Followed by, the potential of circHIPK3 as biomarkers for prognosis and chemoresistance is confirmed. These findings not only shed light on the molecular basis of circHIPK3, but also lay the groundwork for future therapeutic strategies.

Based on which, we would like to propose some dynamic future research directions. Firstly, to obtain a more accurate relationship between circHIPK3 with other traditional categories like stage, grade, or prognostic indexes like OS, PFS and so on, we need to enlarge the sample size as much as possible. In addition, it might also be incorporated into a risk model with other indicators to predict patient outcomes. Moreover, in view of its essential roles in modulating chemosensitivity, it is necessary to determine whether its combination with first-line chemotherapy drugs is superior for treatment. At the same time, it is crucial to strictly grasp its indications, appropriate course and dose. Meantime, circular RNAs have been identified for their enrichment and stability in exosome recently [42], which can be secreted under different physiological and pathophysiological conditions and detected in the circulation and urine [64]. Accumulating evidences have elucidated that exosomal circHIPK3 plays essential roles in the pathogenesis of diseases including cancers. For instance, exosomal circHIPK3 derived from hypoxia-induced cardiomyocytes (CMs) was reported to promote VEGFA expression by inhibiting miR-29a activity and then accelerated cell cycle and proliferation in cardiac endothelial cells [80]. Followed by, another study demonstrated circHIPK3 was obviously increased in TMZ-resistant glioma cells’ exosomes [25]. Taken together, these results indicated that circHIPK3 in exosomes deserves further attention. Though, due to their low abundance, it seems difficult to detect circHIPK3 in exosomes without an accurate approach and algorithms. Consistently, almost all studies at present only reported the role of circHIPK3 as a miRNA sponge, it remains to discover other mechanisms of it to regulate gene expression.

Due to their unique characteristics such as stability, conservatism, abundance and specificity [30], circRNAs have been regarded as promising diagnostic biomarkers and therapeutic targets. Multiple studies have demonstrated that circRNAs are closely related to tumorigenesis and disease progression in a complex gene regulatory network. Present studies on the molecular mechanism of circHIPK3 are expected to provide a grander prospect in precise genetic target therapy. We believe that a more comprehensive circRNA-miRNA-mRNA regulatory network can be revealed by combining bioinformatics studies with experimental results in vivo and in vitro, so as to confirm the value of circular RNAs as prognostic or chemoresistance biomarkers, and finally apply them to the clinical practice of gene targeted therapy.

## Data Availability

Not applicable.
